# Colorectal cancer survival in Manizales, Colombia, 2008-2017: a population-based study

**DOI:** 10.1590/1980-549720230040

**Published:** 2023-09-18

**Authors:** Eduardo Antonio Guzmán-Gallego, Nelson Enrique Arias-Ortiz, Juan David Rodríguez-Betancourt

**Affiliations:** I Universidad de Caldas, Health Research Institute, Basic Clinical Department – Manizales, Colombia.; II Universidad de Caldas, Health Research Institute, Research group on Health Promotion and Disease Prevention – Manizales, Colombia.

**Keywords:** Colorectal cancer, Survival, Colombia, Prognosis, Cáncer colorrectal, Supervivencia, Colombia, Pronóstico

## Abstract

**Objective:**

To determine 5-year survival in patients with colorectal cancer (CRC) according to patient and tumor characteristics.

**Methods:**

Longitudinal study based on incident cases of invasive CRC between 2008 and 2017 captured by the Manizales Population-based Cancer Registry (n=850). Patients were followed up to August 24^th^, 2021. Cause-specific survival and net survival were calculated for sociodemographic and tumor characteristics, and Cox multivariate was fitted.

**Results:**

Fifty-five percent of cases occurred in women. The most frequent histological type was adenocarcinoma (78.2%). The most frequent locations were rectum (32.0%), ascending colon (16.6%), and sigmoid (16.2%). Twenty-five percent of cases were diagnosed in stage IV. There were 567 deaths due to CRC. The 5-year specific survival was 45.8% (95%CI 42.4–49.3), with independent effects for age (HR=1.83; 95%CI 1.26–2.65 age >75 years *vs*. <50 years) and advanced clinical stage (HR=2.5 and HR 5.7 for stages III and IV *vs*. stage I, respectively). Lower survival was observed in patients of medium socioeconomic status compared with higher socioeconomic status (HR=1.52; 95%CI 1.08–2.14), but not in patients of low socioeconomic status. No independent effects were observed for the health insurance regime.

**Conclusions:**

In Manizales, approximately 5 out of 10 patients with invasive CRC die in the first five years after diagnosis, with a lower survival in patients older than 75 years, from medium socioeconomic level and diagnosed in advanced clinical stages.

## RESUMEN

**Objetivo:**

Estimar la supervivencia a 5 años en pacientes con cáncer colorrectal (CCR) según características del paciente y del tumor.

**Métodos:**

Estudio longitudinal a partir de los casos de CCR invasivo diagnosticados entre 2008 y 2017 captados por el Registro Poblacional de Cáncer de Manizales (n=850). Se realizó seguimiento hasta el 24 de agosto de 2021. Se calculó la supervivencia causa-específica y la supervivencia neta para las características sociodemográficas y del tumor, y análisis multivariado de Cox.

**Resultados:**

El 55,5% de los casos se presentaron en mujeres. El tipo histológico más frecuente (78,2%) fue el adenocarcinoma. Las localizaciones más frecuentes fueron recto (32,0%), colon ascendente (16,6%) y sigmoides (16,2%). El 25,5% de los casos fue diagnosticado en estadio IV. Ocurrieron 567 muertes por CCR. La supervivencia específica a 5 años fue del 45,8% (IC95% 42,4–49,3), con efectos independientes para la edad (HR=1,83; IC95% 1,26–2,65 edad >75 años vs. edad <50 años) y el estadio clínico avanzado (HR=2,5 y HR 5,7 para estadios III y IV vs. estadio I). Se observó una menor supervivencia en pacientes de nivel socioeconómico medio en comparación con el grupo de mayor posición socioeconómica (HR=1,52; IC95% 1,08–2,14), pero no en los pacientes de posición socioeconómica baja. No se observaron efectos independientes para el régimen de aseguramiento en salud.

**Conclusiones:**

En Manizales, aproximadamente 5 de cada 10 pacientes con CCR invasivo fallecen en los primeros cinco años del diagnóstico, con sobrevida menor en pacientes mayores de 75 años, de nivel socioeconómico medio y diagnosticados en estadio clínico avanzado.

## INTRODUCTION

Colon cancer is the tumor developed due to malignant degeneration of the cells of the large intestine, from the ileocecal valve to the rectosigmoid flexure; from the latter to the anus, it is called cancer of the rectum^1^, and together they are called colorectal cancer (CRC). Between 85-90% of cases are diagnosed after the age of 55; however, it can affect the entire population^2^.

In Latin America, the incidence of all types of cancer, and in particular CRC, has increased mainly due to the increase and aging of the population and changes in eating habits and physical activity^3^.

According to Globocan, 10,873 new cases of CRC were estimated in Colombia in 2020, for an age-adjusted incidence rate (AAIR) of 16.9 per 100,000 inhabitants; and 5,417 deaths, corresponding to a rate of 8.2 deaths per 100,000 inhabitants^4^. Population-based studies in Bucaramanga^5^, Cali^6^, and Manizales^7^ have shown an increasing trend in the incidence and mortality from this cancer.

Population-based studies that provide data on survival from this cancer in Latin American populations are scarce. Manizales, Colombia, is an intermediate city with a degree of development similar to that of other Latin American populations and has a population-based cancer registry that meets the standards proposed by the International Agency for Research on Cancer (IARC)^8^. The objectives of this work were: to describe the characteristics (of both patients and tumor) in the incident cases of CRC; to estimate cause-specific survival and net survival at one, three, and five years after diagnosis; and to evaluate CRC survival predictors.

## METHODS

### Type of study and participating population

Longitudinal, retrospective, analytical, population-based study carried out in Manizales, Colombia, an Andean city with 434,403 inhabitants, according to the 2018 National Household and Population Census^9^.

### Case data

The Manizales Cancer Population Registry (*Registro Poblacional de Cáncer de Manizales* – RPC-M) gathers information on all primary malignant tumors through active search for data on tumor and patient characteristics in the records of health care providers, in accordance with IARC rules. Topographic and morphological coding of the tumors follow the standards of the International Classification of Diseases for Oncology, third edition (ICD-O-3). From the RPC-M database, all incident cases were identified between January 1^st^, 2008 and December 31^st^, 2017, whose topographic codes corresponded to the locations of the colon (C18x), rectosigmoid junction (C19), and rectum (C20); the cases correspond to a census of all CRC cases diagnosed in Manizales during the study period. Five cases were excluded in which the review of pathology and RPC-M records found to correspond to other diagnoses: one primary case of ovary tumor, one case of haematological neoplasia; two primary cases of stomach tumor; and one case located in the anus; One case diagnosed by death certificate only (DCO) was found, which was excluded from the analysis since the follow-up time was equal to zero.

CRC histologic subtypes and topographic sites were defined according to ICD-3 groupings. The data necessary for cancer staging were obtained from the patients’ clinical histories and pathology reports; The criteria of the 7^th^ edition of the American Joint Committee on Cancer, the classification in use during the study period, were applied. Each patient was included only once in the analysis, that is, diagnoses of metachronous or synchronous tumors were not taken into account, but only the first diagnosis of CRC was included.

The incidence data generated by the RPC-M for the periods 2003-2007 and 2008-2012 have been included in volumes X and XI of the book Cancer Incidence in Five Continents; the data for the period 2013-2017 were recently accepted and are in the process of being published in volume XII. Indicators of good data quality include morphological verification in 99% of the cases; percentage of missing data less than 15% (except for staging); and percentage of follow-up losses of 0.82% of the cases.

### Health insurance regime

The health insurance system in Colombia covers 97.7% of the population (cut-off year 2020). There are different health insurance regimes depending on the income and employment situation of the population: contributory (workers and their families, 73.5% of the population of Manizales); subsidized (vulnerable populations, 18.2%); and special and exceptional (workers in the education sector, police, and military, 2.4%). The proportion of people without health insurance in Manizales is considered low (5.9%)^10^. Health insurance data of the cases were obtained from the medical records.

#### Socioeconomic level

In Colombia, socioeconomic stratification is a physical classification of residential properties and their surroundings, from 1 to 6, where stratum 1 corresponds to the poorer population and stratum 6 to the wealthier population; the stratum reflects the socioeconomic conditions of the families and defines the collection of taxes and contributions, and the allocation of subsidies^11^.

#### Causes of death

Death certification coverage in Manizales is considered exhaustive (approximately 99%). All deaths are certified by a physician; the accurate certification rate is 96.4% for total deaths and 93.2% for cancer deaths^12^.

#### Follow-up

Passive follow-up was performed for 60 months or until August 24^th^, 2021 to identify the event (death from colorectal cancer or from other causes) and the time to the event, based on the date of incidence defined according to the IARC rules for population-based cancer registries. Using the personal identification number, the vital status of each case was confirmed by consulting government databases (electoral census and health insurance records). Then, the date and cause of death of the patients identified as deceased in the official death certificates were verified through the vital statistics platform of the National Administrative Department of Statistics (*Departamento Administrativo Nacional de Estadística* – DANE) with the support of the departmental health authority. Patients who did not experience the event at the end of follow-up were censored. Loss to follow-up was 0.82% (n=7 cases), and lost cases were treated as censored on the date of last known contact.

#### Statistical analysis

One-, three-, and five-year cause-specific survival probabilities (for each patient or tumor characteristic) were calculated using the Kaplan-Meier functions; the log-rank test was used for comparisons. Three Cox regression models were fitted to estimate the effects of the characteristics of interest:

1. A univariate model (null model);
2. “Model A” that includes insurance or socioeconomic level (one variable at a time) adjusted for the other characteristics (age, gender, topography, morphology, stage); and
3. “Model B” that includes both characteristics, insurance, and socioeconomic level, in addition to the adjustment characteristics already mentioned.

The proportional hazards assumption was tested with the Schoenfeld residual test. The final equation of the model was:


$$\begin{array}{l} \ln(\mathrm{HR})=\ln\left(\lambda_{0}(t)\right)+\beta_{1}*\text{insurance}+\beta_{2}*\text{socioeconomic}\;\text{level}+\beta_{3}*\text{gender}+\beta_{4}*\text{age}\;\text{range}+\beta_{5}*\text{topography}+\beta_{6}*\text{histology}+\beta_{7}*\text{stage} \end{array}$$

For purposes of comparability with other studies, net survival was calculated using the Perme et al.^13^ estimator, adjusting for age according to the weightings suggested by the International Cancer Survival Standards indicated by García et al.^14^ Statistical analysis was performed in Stata 14.2^®^ (StataCorp LLC, College Station, TX, USA).

### Ethical considerations

This research was approved as minimal risk by the Institutional Research Ethics Committee of the School of Health Sciences of *Fundación Universitaria del Área Andina* (minute No. 14 of July 13^th^, 2022).

In Colombia, notification is mandatory for some cancers since 2015, but not for CRC. Data collection was carried out after the sources that voluntarily provide their data to the RPC-M gave their institutional informed consent, guaranteeing the confidentiality of the information in accordance with the guiding principles of the IARC. This study conforms to the Strengthening the Reporting of Observational Studies in Epidemiology (STROBE) guidelines^15^.

## RESULTS

A total of 850 incident cases of CRC in the study period were analyzed. The sociodemographic characteristics of the patients, and the histological and morphological description of the tumors are presented in Supplementary Table 1. 55.5% of the cases were women. The mean age at diagnosis was similar between men and women; 57.1% of the cases were people between 50 and 74 years of age; the distribution of cases according to age range was similar in both genders.

With regard to insurance, most of the patients were affiliated to the contributory regime, with no differences according to gender. Regarding the socioeconomic level, 67.1% of the patients belonged to the middle class. 99.8% (n=851) of the cases had a microscopic diagnosis. For both genders, the most frequent histological subtype (69.3%) was adenocarcinomas. 32% of the cases were located in the rectum. Half of the cases were diagnosed in stages III and IV.

CRC-specific survival according to study characteristics is shown in Supplementary Table 2. At 5 years after diagnosis, cause-specific survival was 45.8% (95%CI 42.4–49.3) and net survival was 49.3% (95%CI 45.0–53.5), with no differences between the periods 2008–2012 and 2013–2017. People older than 75 years presented a cause-specific survival 17 percentage points lower than that of people between 50 and 74 years, and 23 percentage points lower than that of the population <50 years (Graphic 1, log-rank=39.3; p=0.0001).

**Graphic 1:**
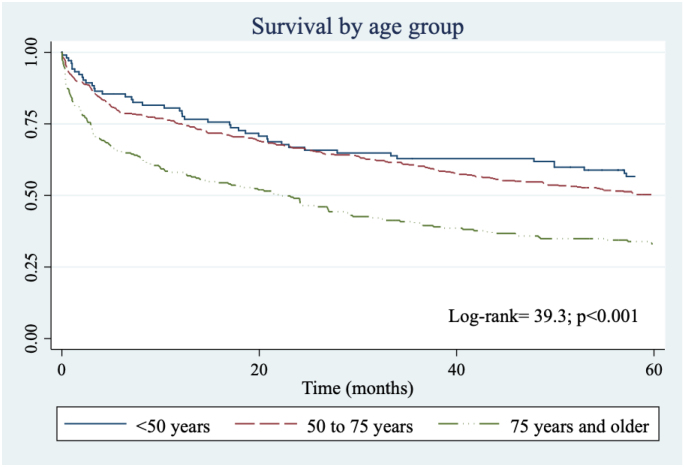
Survival according to age range.

The health insurance regime does not seem to influence the survival of these patients (log-rank=3.22; p=0.20). Neither were significant differences observed in cause-specific survival according to gender (log-rank=1.27; p=0.26). Although a survival of about 10 percentage points less is observed in patients of low and medium socioeconomic status compared to those of high status, when applying the test excluding cases without information on socioeconomic status, the resulting difference is not statistically significant (log-rank=4.25; p=0.12). Differences in cause-specific survival were observed according to histological subtype in favor of patients with adenocarcinomas compared with other histological types and unspecified tumors. No statistically significant differences were observed between colon tumors and rectosigmoid-rectum junction tumors (log-rank =1.89; p=0.17). Regarding the clinical stage of CRC, survival in patients with early diagnosis (stage I) is four times the survival in patients with stage IV (Graphic 2, log-rank=156.5 p<0.001).

**Graphic 2:**
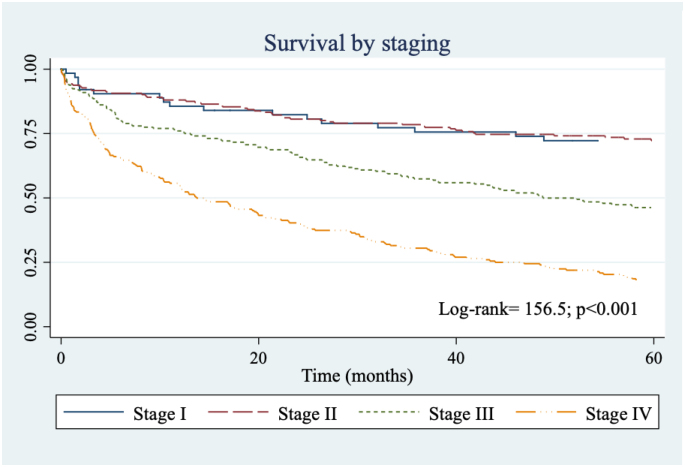
Survival according to staging.

Table 1 presents the results of the univariate and multivariate analyses modeled with Cox regression according to the characteristics of interest. Patients aged 75 years oldqq and older have almost twice the instantaneous risk of dying from CRC before 5 years than patients younger than 50 years (HR=1.97; 95%CI 1.44–2.69). In all three models, a greater instantaneous risk of dying was observed during the 5 years after diagnosis in the group of patients of medium socioeconomic level compared to the group of better social position, but without observing this effect in the group of low socioeconomic level. The clinical stage of the diagnosis behaved as the variable with the greatest weight in survival, with instantaneous risk ratios of 2.5 and more than 5 for stages III and IV compared to patients diagnosed with stage I; such risk is maintained in the multivariate analysis, indicating that the effect of these characteristics is independent of the other characteristics included in the model.

**Table 1:** Multivariate analysis (Cox proportional hazards models) of CRC cause-specific survival according to patient and tumor characteristics. Manizales, 2008–2017.

		Univariate analysis	Multivariate analysis	
		HR (95%CI)	Model A*	Model B†
			HR (95%CI)	HR (95%CI)
Insurance				
	Private/special/exception	Ref	Ref	Ref
	Contributory	1.55 (0.89–2.70)	1.46 (0.77–2.76)	1.28 (0.68–2.41)
	Subsidized	1.70 (0.95–3.10)	1.89 (0.97–3.68)	1.64 (0.84–3.21)
Socioeconomic level				
	High	Ref	Ref	Ref
	Medium	**1.36 (1.01–1.85)**	**1.59 (1.13–2.24)**	**1.52 (1.08–2.14)**
	Low	1.41 (0.96–2.07)	1.52 (0.98–2.34)	1.37 (0.88–2.13)
Gender				
	Male	Ref	Ref	Ref
	Female	1.11 (0.93–1.33)	0.95 (0.77–1.18)	0.99 (0.80–1.22)
Age (years)				
	<50	Ref	Ref	Ref
	50 to 74	1.13 (0.83–1.52)	1.12 (0.79–1.57)	1.06 (0.74–1.50)
	≥75	**1.97 (1.44–2.69)**	**1.91 (1.32–2.74)**	**1.83 (1.26–2.65)**
Topography				
	Colon, SAI	Ref	Ref	Ref
	Rectosigmoid/rectum junction	1.14 (0.94–1.36)	1.14 (0.92–1.41)	1.04 (0.84–1.30)
Histologic subtype				
	Adenocarcinomas	Ref	Ref	Ref
	Other histological subtypes and without specification	1.37 (1.13–1.67)	1.13 (0.90–1.25)	1.00 (0.79–1.26)
Clinical stage				
	I	Ref	Ref	Ref
	II	1.18 (0.1–1.97)	1.16 (0.69–1.96)	1.23 (0.72–2.12)
	III	**2.39 (1.47–3.88)**	**2.34 (1.42–3.87)**	**2.51 (1.50–4.21)**
	IV	**5.24 (3.25–8.43)**	**5.48 (3.35–8.96)**	**5.71 (3.44–9.48)**

* Model A for health insurance: likelihood ratio (LR)=174.12; Schoenfeld test=22.2 p=0.07. Model A for socioeconomic level: LR=163.98; Schoenfeld test=15.1 p=0.37;

† Model B: LR=166.02; overall Schoenfeld test=19.5, p=0.27. Schoenfeld residuals=19.5 p=0.27 for model B; Schoenfeld model A assurance=22.2 p=0.07; Model A for socioeconomic level Shoenfeld=15.1 p=0.37). In bold: statistically significant effect measures.

## DISCUSSION

This investigation represents a report of a cohort of patients diagnosed with CRC in a medium-sized Colombian Andean city with internationally comparable population-based data. Population studies on cancer survival are a useful input for monitoring country policies aimed at disease control^16,17^ and, in this sense, this study constitutes a contribution to the evaluation of policies in Colombia, particularly its 2012–2021 Cancer Control Plan^18^.

The diagnosis of CRC in Manizales has a similar presentation in women and men, and survival is also similar in both genders. The diagnosis is often made in advanced stages of the disease, probably due to the lack of initial alarm symptoms for patients, and the lack of organized early detection programs^19^.

In Colombia, some studies, such as the one by Montenegro et al, reported for Medellín and Neiva lesions mainly in the rectum that coincide with the findings of this study^20^. Contrary to what was reported in other studies in Bucaramanga and Medellín, where the greatest affectation was in women^5,21^, no difference was observed in this study regarding gender. The most affected age group in this study were patients between 50 and 74 years with a similar average for both genders around 65 years, coinciding with data from other researchers in Colombia^22^, possibly in relation to early diagnosis recommendations issued by the Ministry of Health. The present study found that the most frequent tumor locations were the ascending colon, the cecum, and the sigmoid colon, a finding similar to that reported in other population studies^23^; and the most frequent histological type were adenocarcinomas, which also agrees with what was reported by Campo-Sánchez et al.^22^

In this investigation, the 5-year cause-specific survival in Manizales was 45.8% (49.3% for net survival), below other studies in Colombia such as that of Campo-Sánchez et al. with 66.7% for colon cancer and 63.9% for rectal cancer^22^, and also lower than that reported for the United States (SEER registries) with 74% and for Europe (EUROCARE study) with 52.1%^17,23,24^, probably explained by better and faster access to health services in those countries and, therefore, earlier diagnosis.

Although socioeconomic position and its relationship with colon cancer survival have been studied, especially the social disadvantages between groups and the presence of cancer in advanced stages at the time of diagnosis in the poorest population groups due to higher barriers to access to diagnosis^25^, the differences found in this study are not consistent with a socioeconomic disadvantage gradient for the population with a lower socioeconomic position, since the differences were only observed for the “middle class” population compared to the more advantaged population.

In the United States, patients without health insurance are up to 1.7 times more likely to present CRC in advanced stages at the time of diagnosis compared to patients with insurance^26^; however, this investigation found no difference in relation to the type of insurance of the patients, a situation that may be due to the small size of the group of patients in special and exceptional regimes, which leads to estimates with wide confidence intervals, and to the proportion of data missing in this variable which difficult the interpretation of the results.

Clinical stages III and IV were the most frequent in this study, a finding consistent with population studies conducted in the United States and Europe^23^, a situation reported in the literature that suggests a lack of access to early detection programs. In the multivariate analysis, staging behaved as the predictor variable with the greatest weight in survival, a finding consistent with that reported in the literature^23^.

A strength of this study was the fact of working with population-based data with international quality standards^8^ indexed by IARC^27^. It is worth highlighting the effort made in the construction of the staging at the time of diagnosis, since this is a variable that is not routinely collected by population cancer registries in the world; Having this variable made it possible to perform a multivariate analysis that measured the effect of other predictive characteristics of survival regardless of how early or late the diagnosis was.

Unlike what happens in many population-based studies, in this investigation it was possible to access the data on the cause of death recorded in the official information on deaths, which allowed to calculate survival by specific cause. However, as Sarfati, Blakely, and Pearce, and Rachet and Coleman point out, it is possible that there is some degree of misclassification of cause of death that may bias survival. In this regard, a study carried out by the National Institute of Cancerology of Colombia reports that 96.4% of all deaths, and 93.2% of deaths from cancer between 2007 and 2011 were certified without errors in Manizales^12^.

Although relative survival and net survival have traditionally been the approach used in population-based survival studies, cause-specific survival is also a valid measure if accurate cause of death information is available. For greater clarity to the reader and to favor comparability with other population-based studies, this paper reports both cause-specific survival and net survival estimates. It should be noted that no statistically significant differences were observed between these two measures.

A series of limitations were identified in the present study: in some histological and clinical characteristics, a considerable percentage of missing data was detected, which could have affected the estimates. No precise information was available on the degree of histological differentiation of the tumors, which is a variable of prognostic importance and could explain part of the effects found for other predictors. On the other hand, the relatively low frequency of cases and events in some categories of certain characteristics made it necessary to dichotomize them in order to adjust the Cox regression, thereby losing information.

The results of this research point to the need for health education in the community, since the detection of lesions in early stages is key to achieving better results in the population. Likewise, this topic should continue to be a fundamental part of medical education to achieve early detection and proper management of this type of injury.

In Manizales, invasive CRC is a pathology that occurs with similar frequency among women and men. The disease is usually diagnosed at ages between 50 and 70 years. Affected patients belong mainly to the contributory regime and affect all socioeconomic strata. Adenocarcinoma histological tumors are the most frequent. Advanced clinical stages (III and IV) were the most frequent, but there is a significant percentage of cases without sufficient information to build a staging.

In Manizales, approximately 5 out of 10 patients with invasive CRC are alive five years after diagnosis. No significant effects of the health insurance regime on patient survival were found. The predictive characteristics of lower 5-year survival in these patients were age greater than 75 years at the time of diagnosis, diagnosis in advanced stages of the disease, non-specific tumor histology, and belonging to the middle socioeconomic level.

Health authorities in Colombia, as well as in other Latin American countries with a similar degree of development, should consider strategies to strengthen early detection programs. Early detection includes early diagnosis (symptomatic individuals) and screening (asymptomatic individuals). CRC screening is important, but it must be organized and conducted at the population level. Although, under conditions of sufficient resources, it is feasible to think about organized population screening strategies, in low- and middle-income countries it is important to invest first in early diagnosis, organize the line of care, and only then think about organizing population screening.

## Data Availability

Anonymized research data is subject to the confidentiality guidelines of the Manizales Cancer Population Registry and will be available upon request.
