# Supplementary material for: Colorectal cancer survival in Manizales, Colombia, 2008-2017: a population-based study
Source: Rev Bras Epidemiol. 2023 Sep 18;26:e230040. doi: 10.1590/1980-549720230040 (PMC10511025; doi:10.1590/1980-549720230040)
Supplement: Supplementary file 1 [file 1980-5497-rbepid-26-e230040-Suppl01.pdf]

Tabla Suplementaria 1. Características sociodemográficas, histológicas y clínicas de los casos incidentes de CCR según sexo. Manizales, 2008-2017

	Hombres		Mujeres		Total		
	N	%	N	%	N	%	
Todos los casos	378	44.5	472	55.5	850	100.0	
Promedio de edad* (DE)	65,3(14,1)		66,2(13,8)				KS 0,57 p=0,001
Edad (años)							
<50	48	12.7	56	11.9	104	12.2	
50 a 74 años	115	56.9	146	57.2	485	57.1	
≥75	215	30.4	270	30.9	261	30.7	
Aseguramiento							
Contributivo	270	71.4	355	75.2	625	73.5	X^2=2,78
Subsidiado	65	17.2	63	13.3	128	15.1	p=0,42
Excepción/Especial	15	4.0	8	1.7	23	2.7	
No asegurado	0	0.0	3	0.6	3	0.4	
Sin información	28	7.4	43	9.1	71	8.4	
Estrato socioeconomico							
Alto	40	10.6	52	11.0	92	10.8	X^2=2,78
Medio	255	67.5	315	66.7	570	67.1	p=0,42
Bajo	35	9.3	57	12.1	92	10.8	
Sin informacion	48	12.7	48	10.2	96	11.3	
Topografía							
Apéndice	6	1.6	4	0.8	10	1.2	**X^2=28,7**
Ciego	29	7.7	29	6.1	58	6.8	**p=0,004**
Colon ascendente	53	14.0	88	18.6	141	16.6	
Colon descendente	23	6.1	43	9.1	66	7.8	
Colon sigmoide	70	18.5	68	14.4	138	16.2	
Colon transverso	12	3.2	26	5.5	38	4.5	
Linfoma tipo MALT	1	0.3	0	0.0	1	0.1	
Recto	132	34.9	140	29.7	272	32.0	
Sitios contiguous (<1segmento)	0	0.0	1	0.2	1	0.1	
Unión rectosigmoidea	23	6.1	11	2.3	34	4.0	
Ángulo esplénico	5	1.3	11	2.3	16	1.9	
Ángulo hepático	5	1.3	10	2.1	15	1.8	
Sin especificación	19	5.0	41	8.7	60	7.1	
Subtipo histológico							
AdenoCa mucinoso	28	7.4	26	5.5	54	6.4	X^2=3,15
AdenoCa en pólipo	9	2.4	12	2.5	21	2.5	p=0,79
Otros AdenoCa, SAI	266	70.4	323	68.4	589	69.3	
Otros carcinomas	14	3.7	20	4.2	34	4.0	
Otros tumores (sarcoma, melanoma, TNPP)	6	1.6	6	1.3	12	1.4	
Tumor maligno, SAI	53	14.0	82	17.4	135	15.9	
Sin verificación morfológica	2	0.5	3	0.6	5	0.6	
Estadio clínico							
I	30	7.9	33	7.0	63	7.4	X^2=5,72
II	95	25.1	98	20.8	193	22.7	p=1,26
III	98	25.9	113	23.9	211	24.8	
IV	83	22.0	134	28.4	217	25.5	
Sin información	72	19.0	94	19.9	166	19.5	

Fuente: Elaboracion propia

Tabla Suplementaria 2. Estimaciones de supervivencia específica y supervivencia neta para cáncer de colon y recto. Manizales, 2008-2017

			**Supervivencia por causa específica**				**Supervivencia neta ajustada por edad***		
	N	Muertes por CCR	A 1 año (IC 95%)	A 3 años (IC 95%)	A 5 años (IC 95%)	*Log-Rank*	A 1 año (IC 95%)	A 3 años (IC 95%)	A 5 años (IC 95%)
Global*	850	567	70,4 (67,2-73,4)	54,6 (51,1-57,9)	45,8 (42,4-49,3)		70,1 (66,7-73,3)	55,7 (51,8-59,5)	49,3 (45,0-53,5)
Periodo									
2008-2012	360	252	71,4 (66,3-75-8)	54,6 (49,2-59,7)	45,8 (40,4-51,0)	X^2=0,23	70,1 (64,8-74,8)	54,0 (48,2-59,4)	46,9 (40,7-52,8)
2013-2017	490	315	69,7 (65,4-73,6)	54,5 (49,9-58,8)	45,9 (41,3-50,4)	p=0,634	70,2 (65,7-74,4)	57,0 (51,6-62,0)	51,2 (45,2-56,8)
Sexo									
Mujeres	472	248	69,4 (65,0-73,3)	53,0 (55,3-64,2)	44,4 (39,7-48,9)	X^2=1,27	69,2 (64,6-73,3)	53,8 (48,7-58,6)	46,7 (41,3-51,8)
Hombres	378	319	71,7 (66,8-76,0)	56,5 (51,2-61,5)	47,7 (42,4-52,9)	p=0,259	71,6 (66,4-76,2)	58,4 (52,1-64,1)	52,6 (45,5-59,3)
Edad (años)									
<50	104	56	**79,6 (70,4-86,2)**	**62,9 (52,7-71,4)**	**56,6 (46,4-65,6)**	**X^2=39,3**	**79,1 (69,7-85,9)**	**63,2 (52,8-72,0)**	**56,5 (46,0-65,7)**
50 a 74	485	293	**75,0 (70,9-78,7)**	**60,2 (55,6-64,5)**	**50,3 (45,6-54,8)**	**p<0,0001**	**73,2 (68,9-77,1)**	**59,5 (54,7-64,0)**	**50,2 (45,2-55,0)**
≥75	261	218	**58,1 (51,8-63,9)**	**40,4 (34,2-46,5)**	**32,9 (26,9-38,9)**		**60,1 (53,0-66,5)**	**44,8 (36,6-52,6)**	**44,1 (34,3-53,4)**
Aseguramiento									
Excepción/Especial/Particula	31	16	77,2 (58,0-88,4)	66,6 (46,8-80,5)	59,2 (39,3-74,5)	X^2=4,8	71,4 (50,4-84,7)	66,9 (42,1-82,0)	69,2 (36,6-87,9)
Contributivo	624	413	70,1 (67,0-74,2)	54,9 (50,8-58,7)	46,0 (41,9-49,9)	p=0,187	71,1 (67,1-74,7)	57,5 (52,8-61,9)	50,6 (45,5-55,5)
Subsidiado	128	93	73,2 (64,5-80,0)	56,6 (47,4-64,8)	44,4 (35,4(53,1)		69,9 (60,5-77,5)	73,2 (42,9-65,5)	43,2 (32,4-53,6)
No asegurado/Sin informació	67	45	58,5 (45,6-69,4)	42,2 (29,9-54,0)	42,2 (29,9-54,0)		61,4 (47,6-72,5)	41,5 (28,9-53,7)	43,4 (29,9-56,1)
Estrato socioeconomico									
Bajo	92	66	**67,1 (56,4-75,7)**	**50,2 (39,4-59,9)**	**40,3 (30,0-50,4)**	**X^2=34,98**	**69,7 (59,0-78,1)**	**52,5 (40,6-63,1)**	**41,6 (29,6-53,1)**
Medio	570	412	**67,3 (63,3-71,0)**	**50,3 (46,0-54,4)**	**41,1 (36,9-45,2)**	**p<0,0001**	**66,2 (61,9-70,2)**	**50,2 (45,4-54,8)**	**43,5 (38,4-48,5)**
Alto	92	59	**76,7 (66,5-84,2)**	**61,7 (50,7-70,9)**	**50,6 (39,6-60,6)**		**79,1 (69,1-86,2)**	**68,2 (56,3-77,4)**	**57,9 (45,1-68,6)**
Desconocido**	96	30	**86,0 (77,1-91,6)**	**77,1 (67,1-84,5)**	**74,9 (64,7-82,6)**		**86,4 (75,6-92,6)**	**80,8 (66,9-89,3)**	**86,4 (66,2-94,9)**
Topografía									
Colon, SAI	544	353	66,6 (62,5-70,4)	50,4 (46,1-54,5)	42,3 (38,1-46,4)	X^2=0,79	68,8 (64,4-72,7)	56,4 (51,4-61,1)	52,8 (47,1-58,2)
Unión rectosigmoidea / Rect	306	217	69,5 (64,0-74,3)	49,8 (44,1-55,3)	37,4 (32,0-42,8)	p=0,373	72,5 (66,7-77,4)	54,6 (48,2-60,6)	43,2 (36,7-49,5)
Subtipo histológico									
AdenoCa	664	434	71,1 (67,5-74,4)	52,7 (48,8-56,4)	42,1 (38,4-45,9)	**X^2=10,58**	73,3 (69,5-76,7)	57,8 (53,4-61,9)	50,4 (45,5-55,0)
Otras histologias	186	136	55,3 (47,9-62,1)	41,2 (34,1-48,2)	34,7 (27,9-41,6)	**p=0,001**	58,9 (50,6-66,3)	47,6 (38,6-56,0)	45,2 (35,4-54,5)
Estadio clínico									
I	63	25	**85,6 (74,2-92,2)**	**75,6 (62,8-84,5)**	**72,2 (59,2-81,7)**	**X^2=133,2**	**85,8 (72,9-92,8)**	**75,2 (59,8-85,4)**	**74,5 (56,2-86,0)**
II	193	84	**88,0 (82,5-91,9)**	**78,5 (71,9-83,7)**	**71,6 (64,5-77,5)**	**P<0,0001**	**89,2 (82,8-93,3)**	**82,9 (74,2-88,9)**	**80,8 (69,5-88,2)**
III	211	137	**76,0 (69,6-81,3)**	**57,4 (50,3-63,8)**	**46,3 (39,3-53,0)**		**76,6 (69,7-82,1)**	**62,5 (54,3-69,7)**	**54,0 (44,8-62,3)**
IV	217	193	**54,7 (47,8-61,1)**	**30,5 (24,4-36,8)**	**18,0 (13,0-23,7)**		**52,7 (45,7-59,2)**	**30,1 (23,8-36,7)**	**17,3 (12,1-23,3)**
Sin información	166	128	**51,5 (43,6-58,8)**	**35,5 (28,3-42,8)**	**31,2 (24,3-38,4)**		**54,0 (45,1-62,0)**	**40,8 (32,0-49,5)**	**39,3 (30,2-48,3)**

*La supervivencia neta se ajustó por edad con la ponderación por grupos de edad del International Cancer Survival Standard ICSS-1, excepto para las estimaciones de supervivencia neta por grupos de edad
